# Participating in Longitudinal Observational Research on Psychiatric Rehabilitation: Quantitative Results From a Patient Perspective Study

**DOI:** 10.3389/fpsyt.2022.834389

**Published:** 2022-02-04

**Authors:** Lorenz B. Dehn, Martin Driessen, Ingmar Steinhart, Thomas Beblo

**Affiliations:** ^1^Department of Psychiatry and Psychotherapy, Evangelische Klinikum Bethel, Universitätsklinikum OWL of Bielefeld University, Bielefeld, Germany; ^2^Department of Psychology, University of Bielefeld, Bielefeld, Germany; ^3^von Bodelschwinghsche Stiftungen Bethel, Bielefeld, Germany; ^4^Institut für Sozialpsychiatrie Mecklenburg-Vorpommern e. V., University of Greifswald, Greifswald, Germany

**Keywords:** observational study, research participation, psychiatric rehabilitation, burden, evaluation, health service research

## Abstract

**Background:**

Longitudinal observational studies play on an important role for evidence-based research on health services and psychiatric rehabilitation. However, information is missing about the reasons, why patients participate in such studies, and how they evaluate their participation experience.

**Methods:**

Subsequently to their final assessment in a 2-year follow-up study on supported housing for persons with severe mental illness, *n* = 182 patients answered a short questionnaire on their study participation experience (prior experiences, participation reasons, burden due to study assessments, intention to participate in studies again). Basic respondent characteristics as well as symptom severity (SCL-K9) were also included in the descriptive and analytical statistics.

**Results:**

To help other people and curiosity were cited as the main initial reasons for study participation (>85%). Further motives were significantly associated with demographic and/or clinical variables. For instance, “relieve from boredom” was more frequently reported by men and patients with substance use disorders (compared to mood disorders), and participants ‘motive” to talk about illness” was associated with higher symptom severity at study entry. Furthermore, only a small proportion of respondents indicated significant burdens by study participation and about 87% would also participate in future studies.

**Conclusions:**

The respondents gave an overall positive evaluation regarding their participation experience in an observational study on psychiatric rehabilitation. The results additionally suggest that health and social care professionals should be responsive to the expectations and needs of patients with mental illness regarding participation in research.

## Introduction

To improve and develop psychosocial health care and rehabilitation, empirical research is needed. This research relies, for a large part, on information that comes from the people who use social and medical support services. Overall, people with mental health problems seem to have positive attitudes toward psychiatric research and mostly indicate altruistic reasons for participation (1–5). However, patients' willingness to participate in research is influenced by different variables from three major domains (6): Sociocultural and demographic factors (e.g., age, gender); individual experiences and attitudes (e.g., prior research experience, general attitudes toward research), and clinical factors (e.g., diagnosis, severity of illness). Within this framework, participation is also influenced by specific characteristics of the study (6). For instance, in a study with *n* = 763 psychiatric patients, Schäfer et al. (4) found a tendency to approve psychosocial (e.g., rehabilitation, role of the family) rather than biological research topics (e.g., genetics, biological treatments). In addition, “invasive” methods like medication trials achieved the lowest acceptance rate (58%), while the highest willingness to participate was found for studies with questionnaires (91%). Aside such general attitudes toward research, studies have, of course, also focused on the extent to which individuals with mental disorders are affected when participating in research projects. Here it has become apparent, that only a minority of participants in psychiatric research become distressed, but without evidence of longer-term harm (7). Although often considered with particular caution, this is as true for trauma-focused research in people with mental illness (8, 9). Nevertheless, researchers must of course be mindful that rates of adverse reactions might exceed those of community-based samples (10, 11).

For evidence-based research on health services and psychiatric rehabilitation, observational studies play an important role, because randomized controlled trials as the scientific gold standard may not be a feasible option under real-world community settings (12–14). Thus, the present study aims to address (a) what reasons motivate patients to participate in a longitudinal observational study on psychiatric rehabilitation? (b) What are the burdens for the study participants? (c) In how far are reasons and burdens associated with possible moderators such as socio-demographics, prior research experience, symptom severity or diagnostic group?

## Methods

### Study Setting

Given the lack of systematic empirical research on supported housing for non-homeless people with severe mental illness, we conducted an observational follow-up study to compare clinical and functional outcomes of supported housing (SH) vs. residential care (RC) (15). In this prospective study, we included *n* = 257 outpatients with severe mental illness, who were intended to enter SH or RC or had just entered one of these rehabilitation programs. The study project has been approved by the local IRB (University of Muenster Ethics Committee, 2017-149-f-S).

### Study Procedure

The underlying observational study comprised three assessment time points across 2 years: Baseline at study entry (t1), intermediate assessment provided one year later (t2), and final assessment after two years (t3). Asides detailed documentation of socio-demographic and clinical variables at baseline, all three assessments included numerous questionnaires [e.g., Social Functioning Scale (16, 17); Manchester Short Assessment of Quality of Life (18), Symptom Checklist Shortform (19), Client Sociodemographic and Service Receipt Inventory (20, 21), Oxford Capabilities questionnaire—Mental Health (22), Camberwell Assessment of Need Short Appraisal Schedule (23, 24), short interview sections on psychosocial care and clinical course parameters, and a short cognitive skills test [Trail-Making-Test (25)]. Many instruments were available for self-completion. However, if patients pointed out any obstacles in doing so, the questionnaires were read aloud by the interviewers. Overall, a wide range of topics were addressed, e.g., daily living skills, mental health symptoms, social networks, life satisfaction, health care system, experiences with justice and police, drug use or family relationships. In general, the whole assessment procedure took about 60–90 min, depending on the how many breaks were taken or how detailed some questions were answered. In some cases, the assessment session took up to 2 h or was split into two appointments. Subsequent to the last assessment at the third study time point, participants were given a brief questionnaire to retrospectively evaluate their study participation experience (see below, measures).

### Participants

Apart from age 18–69 years inclusion criteria into the underlying observational study were (i) a severe mental illness, diagnosed as defined by ICD-10 by a psychiatrist which (ii) lasted at least 2 years and (iii) was associated with a handicap that constitutes the right for supported housing according to the German social law IX. Exclusion criteria were non-sufficient German speaking and severe physical illness.

Prior to the first study assessment, outpatients were provided with detailed verbal and written study information, and gave their written informed consent to participate in the study. At the beginning of each study assessment, participants were re-informed about the study background and the options to skip parts of the study or to take a break at any time. The participants received a compensation of 10 Euros for the first and 20 Euros for each of the two following assessments.

*N* = 257 individuals were initially included in the observational study, *n* = 56 dropped out before the final assessment, *n* = 15 declined to answer the study participation questionnaire, and *n* = 4 individuals had over 50% missing values. Thus, the present study is based on data from *n* = 182 participants. These enrolled respondents did not significantly differ from the group of dropped out and excluded patients in terms of gender (*p* = 0.780), diagnostic group (*p* = 0.166), psychopathological symptom burden (*p* = 0.733), or housing condition (*p* = 0.330), but they were on average slightly older (*Md* = 41.5, *M* = 41.5/SD = 12.9 vs. *Md* = 35.0, *M* = 37.9/SD = 13.6, *U* = 0.5689.0, *Z* = −2.10, *p* = 0.036).

### Measures

#### Sociodemographic and Clinical Data

Sociodemographic and clinical information (e.g., age, occupational status, clinical diagnosis) was obtained by a structured interview and additional medical information provided by staff members of the psychiatric rehabilitation institutions during the baseline assessment. The burden of psychopathology was assessed via self-report by the 9-item Symptom Checklist [SCL-K-9 (19)], both at the first and the last assessment. We report the SCL-K9 mean score (0–4) as a Global Severity Index [GSI, (26)].

#### Evaluation of Study Participation

Subsequently to the final assessment, 2 years after the start of the index study, participants were asked to provide a retrospective evaluation of their study participation by means of a short questionnaire. To make data collection quick and easy, yes-no questions were favored. Therefore, the applied questionnaire was compiled from single questions taken from existing scales [Hamburg Attitudes to Psychiatric Research Questionnaire (4, 27)] or previous studies, as can be seen in Table 1.

**Table 1:** The evaluation questionnaire.

**Questions/items (+ response options)**	**Origin/sources**
A: Have you ever participated in a scientific study before? *(yes/no)*	Schäfer et al. (4, 27)
B: What have been your reasons for participating in this study?	
B1: I was curious *(yes/no)*	Hall et al. 2018 (5)
B2: To help other people *(yes/no)*	Schäfer et al. (4), Hall et al. (5)
B3: Genuine interest in research *(yes/no)*	Schäfer et al. (4, 27)
B4: To get relieve from boredom *(yes/no)*	Schäfer et al. (4, 27)
B5: To talk about illness *(yes/no)*	Schäfer et al. (4, 27)
B6: Other people wanted me to do so *(yes/no)*	Hall et al. (5); Zullino et al. (1)
B7: Because of the monetary compensation (*yes/no)*	Schäfer et al. (4), Zullino et al. (1)
C1: How burdensome did you find the study assessments? *(not at all, a little bit, moderately, quite a bit, very much)*	Question type and 5-Point-Likert-Scale based on established scales [see (26)]
C2: If “quite a bit” or “very much”: What was most burdening? *(open response option)*	
D: Would you consider taking part in studies in the future? *(yes, no, don't know)*	Tallon et al. (28), Boothyoyd et al. (10)

### Data Analysis

In addition to baseline socio-demographic and clinical sample data, frequencies, and percentages were obtained for all items of the study participation questionnaire. Using the response categories (e.g., yes vs. no), appropriate group comparisons were then performed with respect to possible influencing factors. For this, we selected the following variables according to the three domains from the model by Pfeiffer et al. (6): age and gender (sociocultural and demographic factors); prior research experience (individual experiences and attitudes), diagnosis, symptom severity at study entry and after 2 years, distress due to study participation (clinical factors). Additionally, the type of supported accommodation was also included to control for possible influences by the purpose of the underlying study.

Depending on variable scaling and distribution, parametric (*t*-Test) or non-parametric (Mann-Whitney-Test, Chi^2^-Test) statistical tests were applied. Normal distribution assumption for metric data was checked using Kolmogorov-Smirnov test. Data was analyzed using SPSS Statistics, version 25. The general significance level was set to 0.05 and two-tailed.

## Results

### Participant Characteristics

Demographic and clinical characteristics of the participants are provided in Table 2. The most prevalent primary diagnosis among the sample were substance-related disorders (29.1%), mood disorders (22.5%), and schizophrenic disorders (19.2%). However, about 75% of the participants also had comorbid psychiatric diagnoses, and about two-thirds had additional (chronic) physical impairments or acute somatic conditions. As could be expected, the average burden of psychopathological symptoms in the participants at baseline and after 2 years was about two standard deviations above the values for a representative population sample [Global Severity Index/GSI: *M* = 0.41, *SD* = 0.51, *N* = 2,057; (19)], but within the range of increased values typically found in psychiatric inpatients [e.g., GSI: *M* = 1.7, *SD* = 0.83, *N* = 2,727; (26)].

**Table 2:** Demographic and clinical characteristics of the participants.

**Variable**		**% (*n*)**
Gender	Female	41,2 % (75)
Age	Mean (SD)	41.5 (12.9)
	Median (Min-Max)	41.5, 20–68
Type of supported accommodation	Residential care (with staff on site)	38.5 % (70)
	Supported housing (in own apartment)	61.5% (112)
ICD-10 diagnostic category	F1: Substance-related disorders	29.1 % (53)
	F2: Schizophrenic disorders	19,2 % (35)
	F3: Mood disorders	22.5 % (41)
	F4: Anxiety, stress-related and somatoform disorders	10.4 % (19)
	F6: Disorders of personality and behavior	11.5 % (21)
	Other mental and behavioral disorders	7.1 % (13)
Symptom severity (SCL-K9) baseline^a^	Mean GSI-score (SD)	1.5 (1.0)
	Median (Min-Max)	1.4 (0-4)
Symptom severity (SCL-K9) after 2 years^b^	Mean GSI-score (SD)	1.3 (1.0)
	Median (Min-Max)	1.1 (0-4)

a *n = 178,*

b *n = 181*.

### Evaluation of Study Participation

#### Total Sample

As can be seen in Table 3, around 71% of all participants indicated that they had never participated in a scientific study before. The main reasons for study participation in retrospect have been to help other people (86.8%), curiosity (85.2%), and a genuine interest in research (73.6%). The reasons “*relief from boredom*” (41.8%) and “*an opportunity to talk about illness*” (48.4%) were overall affirmed by almost one in two. The prospect of monetary compensation for participation played a role for more than one-third of individuals (35.7%), and one in ten felt prompted to participate by others (10.4%). More than half of the whole sample (58.8%) did not feel burdened in any way by participating in the study and its repeated assessment procedures. Only <5% (*n* = 9) of the participants indicated that they had felt “*quite a bit*” or “*very much*” burdened. Of these respondents, *n* = 7 people provided information about what had burdened them the most: too many questions (twice), too long surveys, dealing with uncomfortable issues, questions about sexuality, questions about family, reflecting on one's health status using numbers. At the end of the questionnaire, a large proportion of respondents (87%) indicated that they would like to participate in studies again in the future.

**Table 3:** Subjective evaluation of study participation (%, *n*).

	**Yes**	**No**	**Missing**
**Prior study participation**	23.6% (43)	70.9% (129)	5.5% (10)
**Reasons for participating in the study**			
To help other people	86.8% (158)	11.5% (21)	1.6% (3)
I was curious	85.2% (155)	14.8% (27)	–
Genuine interest in research	73.6% (134)	23.6% (43)	2.7% (5)
To talk about illness	48.4% (88)	47.8% (87)	3.8% (7)
To get relieve from boredom	41.8% (76)	56.0% (102)	2.2% (4)
Because of the monetary compensation	35.7% (65)	62.1% (113)	2.2% (4)
Other people wanted me to do so	10.4% (19)	87.4% (159)	2.2% (4)
**Burden due to study participation (*n* = 181)**			
Not at all	58.8% (107)		
A little bit	24.2% (44)		
Moderately	11.5% (21)		
Quite a bit	4.4% (8)		
Very much	0.5% (1)		
**Would participate in study again (*n* = 180)**	87.4% (159)	1.6% (3)	Don't know: 9.9% (18)

#### Influential Factors

To be consistent with the dichotomous yes-no questions and to compensate group sizes, the response categories for the burden item (not at all vs. a little bit/ moderately/ quite a bit/ very much) and the re-participation question (yes vs. no/don't know) have been dichotomized for the following analyses. Moreover, non-parametric Mann-Whitney tests were used, because of partially unbalanced group sizes and non-normally distributed metric variables in several subgroups (KS-Test: *p* < 0.05).

##### Age

For all evaluation items, there were no differences in age between response categories (*p* > 0.05), except for the participation reason “*Other people wanted me to do so*”. Here, those *n* = 19 participants who said they had initially attended because of others were on average somewhat older, than those who denied this item (Md = 53.0, *M* = 47.4/SD = 14.3 vs. Md = 41.0, *M* = 41.1/SD = 12.6, *U* = 1070.0, *Z* = −2.08, *p* = 0.037).

##### Gender

A significant difference with respect to gender occurred only for the participation reason “*To get relieve from boredom*” (Chi^2^ = 6.33, Phi = −0.189, *p* = 0.014). Here, men (50.5%) were significantly more likely to affirm this reason than women (31.5%).

##### Supported Accommodation Type

The questionnaire responses did not show any significant differences between persons from Residential Care or Supported Housing (*p* = 0.339–1.00).

##### Prior Research Participation

There were no significant differences between persons with and without prior experience in either the various reasons for participation (*p* = 0.107–1.00) nor the level of burden experienced (*p* = 1.00). Among those persons who had never participated in research before, the proportion who would consider a further study participation (90.7%/*n* = 117) did not significantly differ from the group with prior experience (92.9%/*n* = 39, *p* = 0.766).

##### Clinical Diagnosis

Analyses of possible differences by diagnosis were at first based on the existing five distinct ICD-10-diagnosis groups (see Table 2). The items of the evaluation questionnaire did not show any significant differences depending on the diagnostic group (*p* = 0.063–0.931), apart from a tendency regarding the participation reason “*To get relieve from boredom*” (*p* = 0.063). However, this trend became statistically significant (Chi^2^ = 8.35, Phi = 0.257, *p* = 0.015) when the analyses were based solely on the three most frequent diagnosis groups (F1–F3, 71% of the total sample). Subsequent *post-hoc* subgroup analyses with adjusted significance level (0.05/3 = 0.0167) showed the following results: In the substance-use disorders group, it was significantly more often reported to have participated as a relieve from boredom than in the mood disorders group (54.9%/*n* = 28, vs. 25%/*n* = 10, Chi^2^ = 8.24, Phi = −0.301, *p* = 0.005). When comparing the mood vs. schizophrenic disorder group, there was only a slight trend (*p* = 0.088), but no difference appeared between the schizophrenic and substance-related disorders group (45,7%/*n* = 16 vs. 54.9%, *p* = 0.511). Since the participation reason “*To get relieve from boredom*” has also been associated with gender (see above), possible gender-related effects depending on diagnosis group were exploratively examined, but without significant results (*p* = 0.120–1.00).

##### Symptom Severity

The level of psychopathological symptom severity assessed at the last study interview and thus at the same time point as the evaluation questionnaire, showed no associations with prior research experience, the various reasons for participation, participation burden, or further participation interest (*p* = 0.199–0.996). However, when the symptom severity from study entry was taken into account, the following significant relationship emerged: Those respondents who agreed with the participation reason “*To talk about illness*” (*n* = 88/48.4%) had higher baseline symptom severity scores than persons who denied this reason (*Md* = 1.33, *M* = 1.36/*SD* = 0.88 vs. *Md* = 1.72, *M* = 1.74/*SD* = 1.04, *U* = 2892.5, *Z* = −2.35, *p* = 0.018).

##### Burden Due to Study Participation

Not any socio-demographic or clinical variable and none of the different reasons for participation were significantly associated with the level of burden due to participation (*p* = 0.078–1.00). However, among those respondents who felt at least somewhat burdened (*n* = 73), significantly fewer persons were willing to participate in a study again compared with those who felt not burdened at all (80.8 vs. 94.3%, Chi^2^ = 7.96, Phi = 0.206, *p* = 0.007).

## Discussion

Long-term observational studies provide an important basis for empirical research in psychiatric rehabilitation and mental health service care. However, little known about how people with severe mental illnesses evaluate their actual experience of participation in such observational studies.

### General Evaluation and Experienced Burden

The present results from a 2-year observational study reveal an overall positive evaluation outcome with participants favorably responding with respect to their research experience. For instance, while about 71% of the patients had never participated in a study before, at the end almost 90% indicated that they would consider participating in future research. This proportion of 90% roughly corresponds to the 86% from a study on managed care in severe mental illness (10), but it was even somewhat higher than the 75% from a RCT on antidepressant medication (28). Moreover, in our study this proportion did not differ between persons with and without previous research experience, so it can be concluded that even patients participating in research for the first time ever gained a good impression from their experiences in a longitudinal observational study. In their paper “‘*That was helpful... no one has talked to me about that before': Research participation as a therapeutic activity*,” Lakeman et al. (29) argued that psychiatric research participation involves processes that are frequently therapeutic in nature or often benefit participants, e.g. telling and retelling details about an aspect of one's life to a researcher.

The majority of participants was not affected by the repeated comprehensive study assessments and did not feel burdened by them at all. Only 5% reported feeling quite a bit or very much burdened. Despite different scaling and reverse polarity, these results are largely consistent with data form an earlier study by Boothyroyd (10). At that time, *n* = 523 participants with a severe mental illness were asked to rate their overall research experience after participating in a 12-months managed care study. While 96% of the respondents indicated that it was a (very, somewhat, slightly) positive experience, 4% perceived it as a (slightly, somewhat, very) negative experience. Furthermore, Jorm et al. (7) have performed a systematic review on whether participation in studies that involved (questionnaire) assessment of symptoms, prevalence or risk factors of psychiatric disorders causes distress. The reviews main conclusion was that only “*a minority of participants (generally*<*10 %) experience distress*” (p. 919).

In our study, the participation burden was neither related to socio-demographic nor to clinical variables, like diagnosis or symptom severity. Although participation-related distress has been found to be associated with poorer mental health and more symptom severity (7), our results are in line with other studies, that found no association between study distress and psychopathology (9, 30). Thus, the present findings may indicate that longitudinal observational research in the field of psychiatric rehabilitation can be conducted with a variety of patients with diverse clinical (and socio-demographic) characteristics. Nonetheless, our results also showed that there might remain a risk that persons who felt some form of burden from research participation will not enroll in future studies. Even if this only refers to rather few cases, this finding highlight that (negative) previous experiences with research can have an impact on future study participation [see also (31, 32)].

### Reasons for Participation and Their Moderators

Consistent with other research, our results showed that the most frequently reported reason for initial study participation was to help other people [e.g., (2, 27, 33)]. Although motivation for study participation is a multi-dimensional construction (6), altruism has been identified to be one of the most relevant factors in medical research settings [e.g., (34, 35)].

In the present study, the least frequent indicated reason for research participation was “*Others wanted me to do so*.” It is notable here, that those few people who cited this reason were slightly older than the rejecters. It could be possible, that older people may have felt more urged to participate, because older age in psychiatric patients has not only been found to be associated with a worse understanding of clinical trial proposals (36), but also with a more frequent refusal of research participation (37, 38). However, these results are somewhat limited because the respondents in the present study were also somewhat older overall than those who were not enrolled in the retrospective evaluation (see Participants).

With respect to the other reasons for participation, curiosity, to help others, research interest, and monetary compensation showed no correlations with one of the influencing variables examined. However, “*to get relieve from boredom*” was associated with male gender and having substance use disorders (as compared to mood disorders). A higher prevalence of boredom experience among men than woman has also been confirmed by large community-based studies (39–41). Moreover, for in- and outpatients with different mental illnesses the experience of boredom is common and of particular relevance to their quality of life (42–45). Thus, distraction from boredom was also cited equally often as a hypothetical reason for research participation by patients with depression and schizophrenia (4). Our results confirm this finding, but additionally point to the relevance of boredom distraction as a salient reason for research participation in substance-use disorder patients. Distracting boredom plays a prominent role in managing symptoms of illness for these patients, as boredom has been identified being among the most common aversive experiences linked to withdrawal symptoms and relapse reasons (46–48).

Finally, those persons who agreed with the reason “*to talk about my illness*” had higher symptom severity ratings at study entry than those who disagreed. Here, one can come to the conclusion that the possible benefit of being able to talk about the illness might have prompted initially more severely ill individuals to participate in the observational study. This could possibly related to social isolation and feelings of loneliness that are common among people with severe mental illness (49) and can be of major concern even under supported housing settings (50). Data form a recent clinical study has shown, that more severe psychopathological symptoms in persons with a mental illness are correlated with greater loneliness, even when objective social isolation, socio-demographic and clinical confounders were controlled for (51). Although we did not assessed loneliness here, the present results can be interpreted in the sense that the greater the symptom burden, the greater the loneliness, and thus participating in a psychosocial research study opens up an occasion to talk to someone “from outside” about one's own mental health problems. This is supported by an interview-study by Woodall et al. (52), in which the prospect of talking to other people about their experiences has been found to act as a facilitator for research participation in people with a first-episode psychosis.

### Implications for Research Practice

The present results have shown that even when participating in a longitudinal observational study with repeated and comprehensive psychosocial assessments, only a minority of participants become distressed, thus strengthening empirical findings from previous studies (7, 53). This is of particular importance because during the initiation of psychiatric studies the appropriateness of the research is often questioned with respect to the vulnerability of the targeted patients (54). For instance, in their systematic review on the experiences of vulnerable people participating in research on sensitive topics, Alexander et al. (55) have concluded that although “*there is little evidence of harm to participants… researchers frequently experience obstacles and the phenomenon known as “gatekeeping” when attempting to conduct research amongst vulnerable populations*” (p. 1). Howard et al. (56) identified paternalism as one of the major recruitment difficulties during an RCT of supported employment for people with severe mental illness. Through interviews, the authors were able to figure out, that mental health care coordinators were focusing more on their perception of patient needs than providing patients with the opportunity to decide whether they would like to participate in research. Therefore, the authors have concluded that due to such paternalistic attitude patients were often denied access to research trials (56). Hughes-Morley et al. (57) have conducted a meta-synthesis on factors affecting recruitment into depression trials, and they identified a “protecting the vulnerable patient” theme among the literature. This means, from the perspective of professional gatekeepers, patients with depression were typically seen as vulnerable, with the need to be protected against an additional burden due to research participation. However, the present findings clearly show that the majority of participants do not feel burdened by the repeated comprehensive study assessments. Moreover, the results suggest, that longitudinal observational research can be conducted with psychiatric outpatients with various clinical and socio-demographic characteristics. In addition to general motives such as altruism and curiosity, research participation depends on certain clinical characteristics. For instance, reducing boredom through study participation might be of particular relevance for substance-use disorder patients, and being able to talk about one's illness appears to be a motivator for patients with more severe symptoms. Thus, honorable and well-intentioned, paternalistic gatekeeping and overprotection by health care professionals could hinder patients from fulfilling their personal motives for research participation (55). Besides such barriers for individual participation, this may also lead to constraints in scientific outcomes. If patients are withheld from study participation because of overestimated negative effects, this can impact data variance and thus weaken the strength of empirical results.

### Limitations

Instead of assessing motives for a hypothetical willingness to participate in psychiatric research (1, 2, 4), the present study asked in retrospect about the reasons for participating in a longitudinal rehabilitation study. However, this comes along with the constraint, that such retrospective assessments may be imprecise due to recall bias (58). Thus, the reasons mentioned for participation might not necessarily have been the initial motives, but were rather influenced by the study experience. If questions on participation reasons would already be implemented at study baseline, then this could not only allow comparison with later retrospective questions on participation reasons, but might also offer interesting insights for detailed analysis of drop-outs. Another limitation of the present study is that the results were gathered as part of a research project on supported housing for people with mental disorders. Although these housing conditions of the index study did not appear to have affected the target parameters, the results should nevertheless be validated in other areas of social psychiatric rehabilitation, such as work, leisure time, or social participation. Moreover, future studies should also be more differentiated in assessing the burden of study participation, instead of just asking about “burden” in general, as it was done in the present questionnaire. For instance, Wenemark et al. (59) have identified five categories of respondent burden in health-related surveys: Cognitive burden (e.g., difficult to understand), unnecessary work (e.g., repetitive questions), distrust (e.g., manipulation), offending questions (e.g., too personal), and distress (e.g., worry, sadness). In addition, a parallel rating by care coordinators regarding the potential burden of study participation on their patients, would allow further interesting analysis of self- and proxy perceptions on this issue. Last but not least, future studies should also ask in more detail about potential benefits from study participation.

## Conclusions

Outpatients with a severe mental illness gave an overall positive evaluation regarding their participation in a 2-year observational rehabilitation study. The majority reported no burden associated with the repeated comprehensive psychosocial assessments, and there was a high willingness to participate in studies again. Altruism and curiosity were cited as the most important reasons for participation in retrospect, and some of the participation motives were associated with socio-demographic and clinical variables. All in all, the burdens of research participation for patients with mental illness should not be overestimated by health and social care professionals. This is not only important in terms of adequately addressing the patients' needs, but also for generating valid scientific results.

## Data Availability Statement

The dataset presented in this article is not readily available due to formal data protection reasons, the anonymized dataset of the present study is only available from the corresponding author on reasonable request. Requests to access the dataset should be directed to Lorenz B. Dehn, lorenz.dehn@evkb.de.

## Ethics Statement

The studies involving human participants were reviewed and approved by University of Muenster Ethics Committee, 2017-149-f-S. The patients/participants provided their written informed consent to participate in this study.

## Author Contributions

LD conceived and planned the research project, was involved in data collection, performed the statistical analyses, and drafted the original manuscript. MD supervised the study project and participated in manuscript preparation. IS was the project leader of the supported housing study and reviewed the article for publication. TB has oversight for the research activity, and critically reviewed the manuscript draft. All authors read and approved the final manuscript.

## Funding

This study on supported housing was supported by a grant from the Stiftung Wohlfahrtspflege NRW (Welfare Foundation North Rhine-Westphalia). The authors confirm, that this foundation had no influence on study design, data interpretation or writing the manuscript. We acknowledge the financial support of the German Research Foundation (DFG) and the Open Access Publication Fund of Bielefeld University for the article processing charge.

## Conflict of Interest

The authors declare that the research was conducted in the absence of any commercial or financial relationships that could be construed as a potential conflict of interest.

## Publisher's Note

All claims expressed in this article are solely those of the authors and do not necessarily represent those of their affiliated organizations, or those of the publisher, the editors and the reviewers. Any product that may be evaluated in this article, or claim that may be made by its manufacturer, is not guaranteed or endorsed by the publisher.
